# Supplementary material for: Camel nanobody-based B7-H3 CAR-T cells show high efficacy against large solid tumours
Source: Nat Commun. 2023 Sep 22;14:5920. doi: 10.1038/s41467-023-41631-w (PMC10517151; doi:10.1038/s41467-023-41631-w)
Supplement: Supplementary file 1 — Supplementary Information [file 41467_2023_41631_MOESM1_ESM.pdf]

Camel nanobody-based B7-H3 CAR-T cells show high efficacy against large solid tumours

Dan Li[1], Ruixue Wang[1], Tianyuzhou Liang[1], Hua Ren[1], Chaelee Park[1], Chin-Hsien Tai[1], Weiming Ni[2], Jing Zhou[2], Sean Mackay[2], Elijah Edmondson[3], Javed Khan[4], Brad St Croix[5], Mitchell Ho[1]

[1]*Laboratory of Molecular Biology, Center for Cancer Research, National Cancer Institute, Bethesda, Maryland 20892, USA*

[2]*IsoPlexis Corporation, Branford, Connecticut 06405, USA*

[3] *Molecular Histopathology Laboratory, Frederick National Laboratory for Cancer Research, Frederick, Maryland 21702, USA*

[4]*Genetics Branch, Center for Cancer Research, National Cancer Institute, Bethesda, Maryland 20892, USA*

[5]*Mouse Cancer Genetics Program, Center for Cancer Research, National Cancer Institute, Frederick, Maryland 21702, USA*

Short Title: Camel nanobody-based B7-H3 CAR-T cells

[*]**Correspondence:** Mitchell Ho, Ph.D., Laboratory of Molecular Biology, National Cancer Institute, National Institutes of Health, 37 Convent Drive, Room 5002, Bethesda, MD 20892-4264, USA. Phone: (240) 760-7848; E-mail: homi@mail.nih.gov

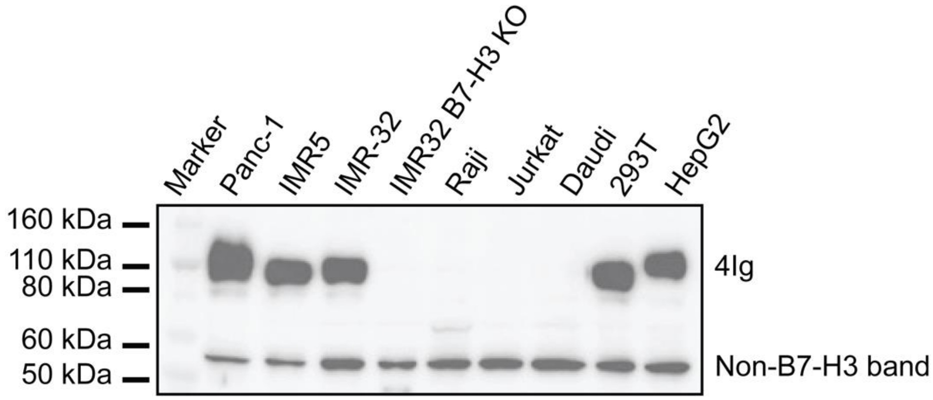

Supplementary Fig. 1: Western blot of B7-H3 expression in B7-H3^{+} and B7-H3^{-} cell lysates using anti-B7-H3 mAb (Abcam; #ab226256). Source data is provided as a Source Data file. n = 3 independent experiments.

```
                         Signal peptide (1-28 aa)               IgV (29-139 aa)
Mouse 2IgB7-H3 MLRGWGGPSVGVCVRTALGVLCLCLTGAVEVQVSEDPVVALVDTDATLRCSFSPEPGFSL  60
Human 4IgB7-H3 MLRRRGSPGMGVHVGAALGALWFCLTGALEVQVPEDPVVALVGTDATLCCSFSPEPGFSL  60
Human 2IgB7-H3 MLRRRGSPGMGVHVGAALGALWFCLTGALEVQVPEDPVVALVGTDATLCCSFSPEPGFSL  60
               *** .*.*.:** * :***.* :*****:****.********.***** ***********

Mouse 2IgB7-H3 AQLNLIWQLTDTKQLVHSFTEGRDQGSAYSNRTALFPDLLVQGNASLRLQRVRVTDEGSY  120
Human 4IgB7-H3 AQLNLIWQLTDTKQLVHSFAEGQDQGSAYANRTALFPDLLAQGNASLRLQRVRVADEGSF  120
Human 2IgB7-H3 AQLNLIWQLTDTKQLVHSFAEGQDQGSAYANRTALFPDLLAQGNASLRLQRVRVADEGSF  120
               *******************:**.******:**********.*************:****:
                                                       IgC (145-238 aa)
Mouse 2IgB7-H3 TCFVSIQDFDSAAVSLQVAAPYSK------------------------------------  144
Human 4IgB7-H3 TCFVSIRDFGSAAVSLQVAAPYSKPSMTLEPNKDLRPGDTVTITCSSYQGYPEAEVFWQD  180
Human 2IgB7-H3 TCFVSIRDFGSAAVSLQVAAPYSK------------------------------------  144
               ******.**.**************

Mouse 2IgB7-H3 ------------------------------------------------------------  144
Human 4IgB7-H3 GQGVPLTGNVTTSQMANEQGLFDVHSILRVVLGANGTYSCLVRNPVLQQDAHSSVTITPQ  240
Human 2IgB7-H3 ------------------------------------------------------------  144
                                  IgV (243-357 aa)
Mouse 2IgB7-H3 ------------------------------------------------------------  144
Human 4IgB7-H3 RSPTGAVEVQVPEDPVVALVGTDATLRCSFSPEPGFSLAQLNLIWQLTDTKQLVHSFTEG  300
Human 2IgB7-H3 ------------------------------------------------------------  144

Mouse 2IgB7-H3 ------------------------------------------------------------  144
Human 4IgB7-H3 RDQGSAYANRTALFPDLLAQGNASLRLQRVRVADEGSFTCFVSIRDFGSAAVSLQVAAPY  360
Human 2IgB7-H3 ------------------------------------------------------------  144
                                  IgC (363-456 aa)
Mouse 2IgB7-H3 --PSMTLEPNKDLRPGNMVTITCSSYQGYPEAEVFWKDGQGVPLTGNVTTSQMANERGLF  202
Human 4IgB7-H3 SKPSMTLEPNKDLRPGDTVTITCSSYRGYPEAEVFWQDGQGVPLTGNVTTSQMANEQGLF  420
Human 2IgB7-H3 --PSMTLEPNKDLRPGDTVTITCSSYRGYPEAEVFWQDGQGVPLTGNVTTSQMANEQGLF  202
                 **************: ********.*********:*******************.***

Mouse 2IgB7-H3 DVHSVLRVVLGANGTYSCLVRNPVLQQDAHGSVTIT  238
Human 4IgB7-H3 DVHSVLRVVLGANGTYSCLVRNPVLQQDAHGSVTIT  456
Human 2IgB7-H3 DVHSVLRVVLGANGTYSCLVRNPVLQQDAHGSVTIT  238
               ************************************
```

Supplementary Fig. 2: Sequence alignment of the ectodomain of human 4IgB7-H3, human 2IgB7-H3, and mouse 2IgB7-H3. The leader peptide is indicated by a black solid line; IgV- and IgC-like domains are indicated by red and green lines. The sequence of peptide #10 (QRVRVADEG**SFTCFVSIR**) and peptide #11 (**SFTCFVSIR**DFGSAAVSL) was highlighted in yellow. The conserved residues are marked with asterisks (*), the residues with similar properties between variants are marked with colons (:), and the residues with marginally similar properties are marked with periods(.).

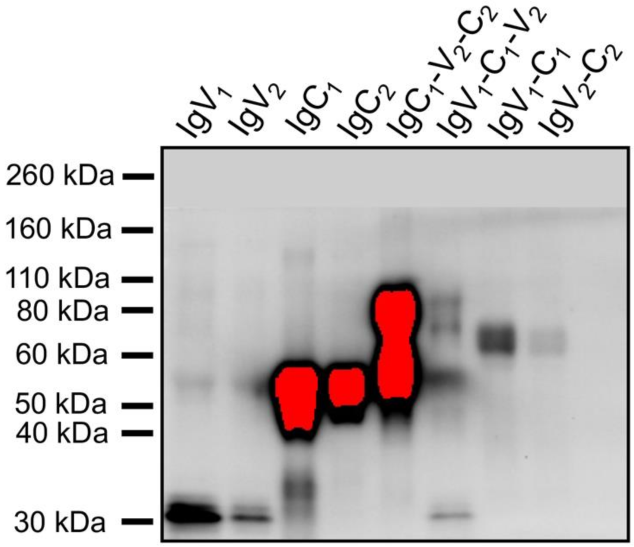

Supplementary Fig. 3: Western blot to examine the interaction between C4-Fc and individual 4Ig fragments. This is the same membrane shown in Fig. 3d with a long exposure of 2 min. $n = 3$ independent experiments.

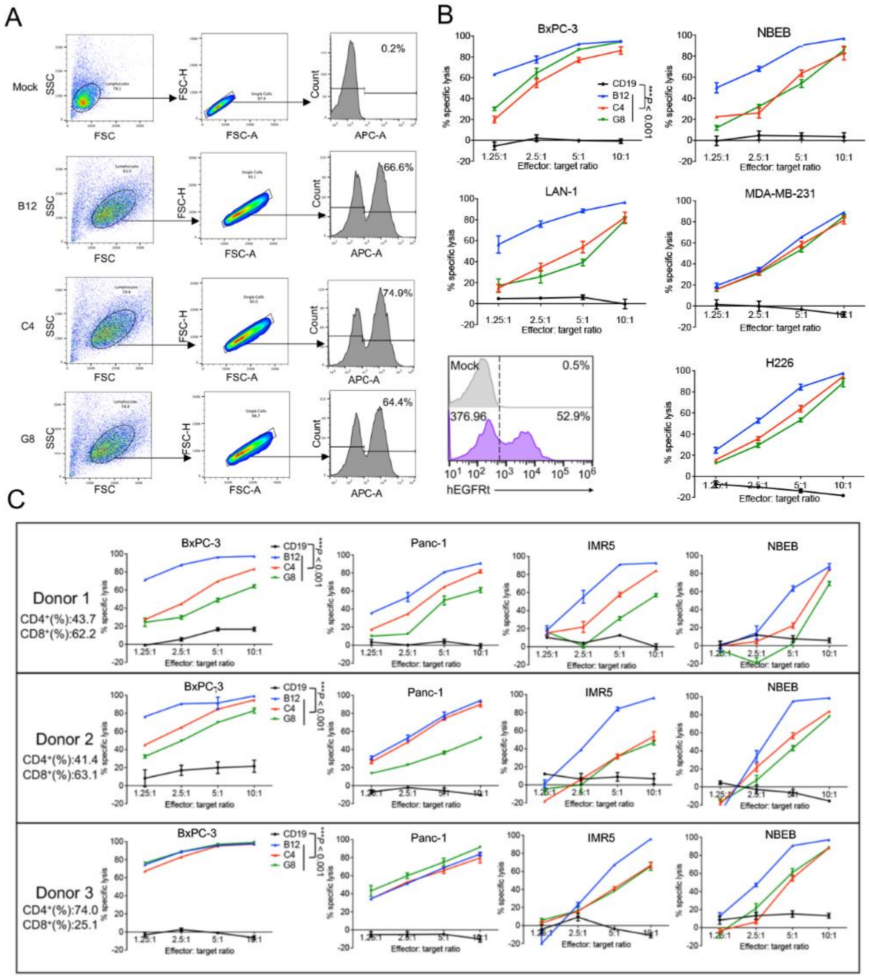

Supplementary Fig. 4: B7-H3-specific nanobody-based CAR-T cells effectively lysed human solid tumor cells *in vitro*. (A) The transduction efficiency of B7-H3 CAR in T cells was examined by hEGFRt expression, including B12(V_HH)-CAR-T, C4(V_HH)-CAR-T, G8(V_HH)-CAR-T, and 376.96(scFv)-CAR-T. Non-transduced T cell was the mock control. (B) Cytolytic activity of B7-H3 nanobody-based CAR-T cells to a human PDAC cell line BxPC-3, two NB cell lines (NBEB and LAN-1), a TNBC cell line (MDA-MB-231), and a LUAD cell line (H226) at various E/T ratios for 24 hours incubation. $n = 3$ independent experiments. Values represent mean ± SEM. $***P < 0.001$, two-tailed unpaired Student's *t* test. (C) Cytolytic activity of three healthy donors-derived B7-H3 nanobody-based CAR-T cells to various tumor cell lines (BxPC-3, Panc-1, IMR5, and NBEB) at various E/T ratios for 24 hours incubation. The percentages of $CD4^+$ and $CD8^+$ CAR-T cells were shown from each donor. The killing data was harvested from three independent experiments. Values represent mean ± SEM. $***P < 0.001$, two-tailed unpaired Student's *t* test. Source data is provided as a Source Data file.

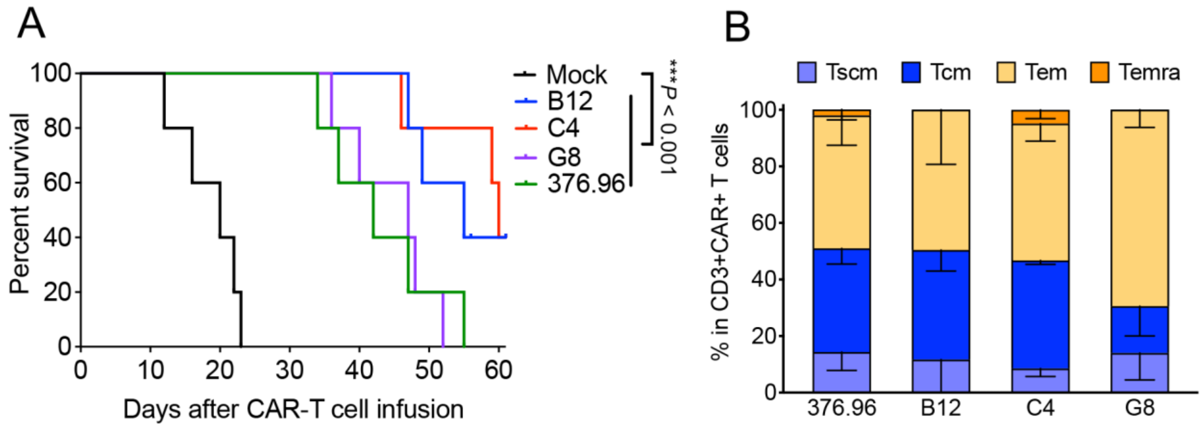

Supplementary Fig. 5: Mice survival and phenotype of circulated B7-H3 CAR-T cells in the CAR-T treated Panc-1-bearing mice. (A) Kaplan-Meier survival curve of mice infused with 5 million B7-H3 CAR-T cells and mock-T cells. *n* = 5 mice/group. ***$P < 0.001$, Log-rank test. (B) Relative proportion of stem cell-like memory (Tscm), central memory (Tcm), effector memory (Tem), and terminally differentiated effector memory (Temra) subsets in circulated $CD3^{+}CAR^{+}$-T cell subpopulations at week 2 in the 2.5 million CAR-T infusion experiment. Statistical analyses are shown from more than three individual samples. Values represent mean ± SEM. *n* = 3 individual sample/group. Source data is provided as a Source Data file.

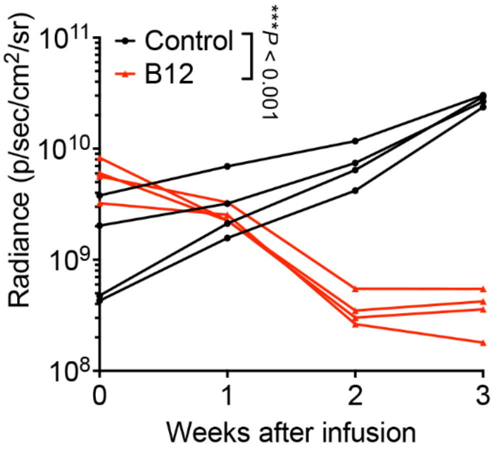

Supplementary Fig. 6: The tumor growth curve of orthotopic Panc-1 tumors in the treatment of B12(V_HH)-CAR-T cells shown in Fig. 5o. $n = 4$ mice/group. Values represent mean ± SEM. ***$P < 0.001$, two-tailed unpaired Student's t test. Source data is provided as a Source Data file.

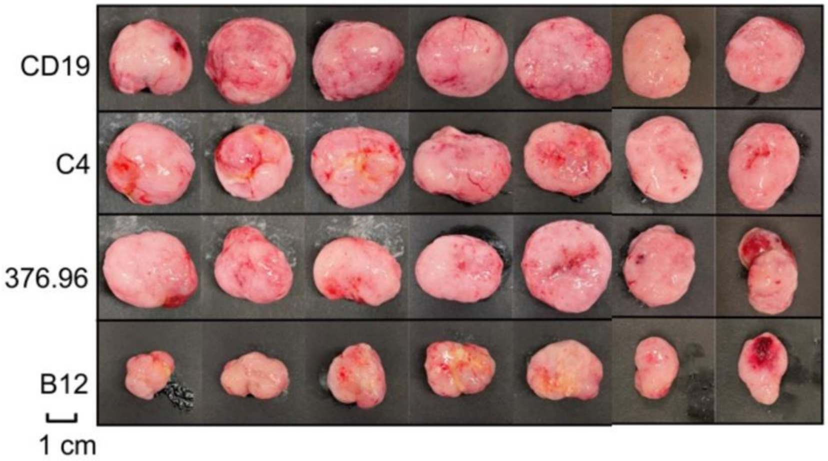

Supplementary Fig. 7: The sizes of NBEB tumors in individual mice from CD19 CAR-T cells and B7-H3 CAR-T cells at the end of the study.

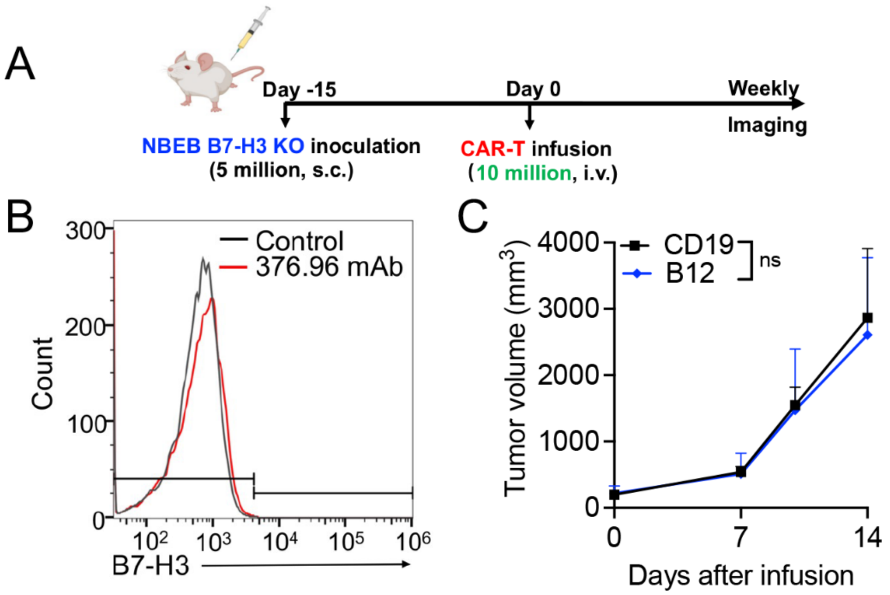

Supplementary Fig. 8: B12(V_HH)-CAR-T cells could not inhibit tumor growth in the NBEB B7-H3 knockout (KO) mouse model. (A) Experimental schema of the subcutaneous (s.c.) NBEB B7-H3 KO mouse model (created with BioRender.com). NBEB B7-H3 KO tumor-bearing NSG mice were i.v. infused with 10 million B12(V_HH)-CAR-T cells or an equivalent number of CD19 CAR-T cells after 15 days of tumor inoculation. *n* = 4 mice/group. (B) Cell surface B7-H3 expression on NBEB B7-H3 KO cells is shown as histograms with the control unstained. The 376.96 mAb was used to examine the expression of B7-H3 on cells. (C) The respective tumor growth curves of two mice groups infused with CAR-T cells. ns, not significant, two-tailed unpaired Student's *t* test. *n* = 4 mice/group. Values represent mean ± SEM. Source data is provided as a Source Data file.

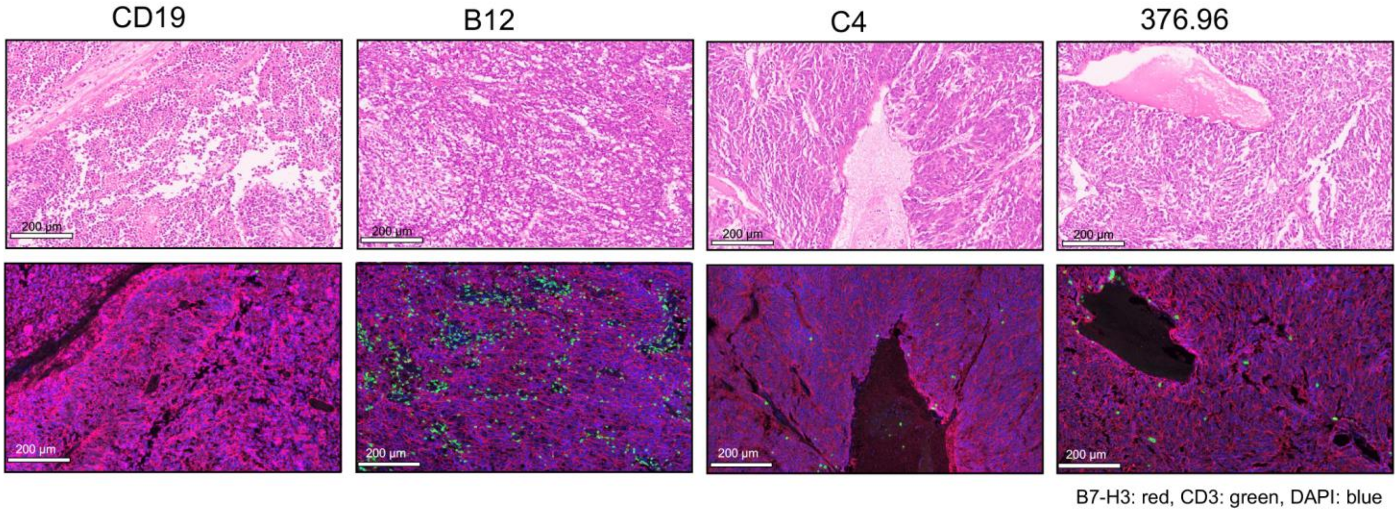

Supplementary Fig. 9: CAR-T cells infiltration in NBEB tumor tissues. The same tumor tissue staining was shown in Figure 6j but with a scale bar of 200 μm. *n* = 3 independent experiments.

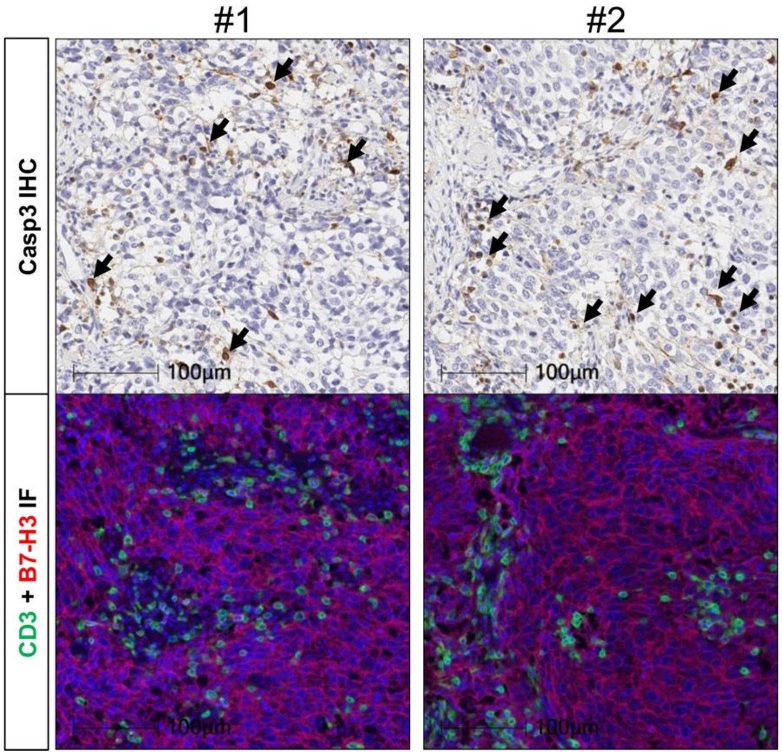

Supplementary Fig. 10: Colocalization of increased apoptotic cells (arrow) stained by Caspase-3 (IHC) and infiltrated B12(V_HH)-CAR-T cells in the NBEB tumor tissues. Two distinct areas (#1 and #2) displaying tumor-infiltrated CAR-T cells were observed. The microscopic examination areas for Caspase-3 IHC staining and CD3+B7-H3 immunofluorescence (IF) staining were precisely matched with each other. Scale bar, 100 µm. n = 3 independent experiments.

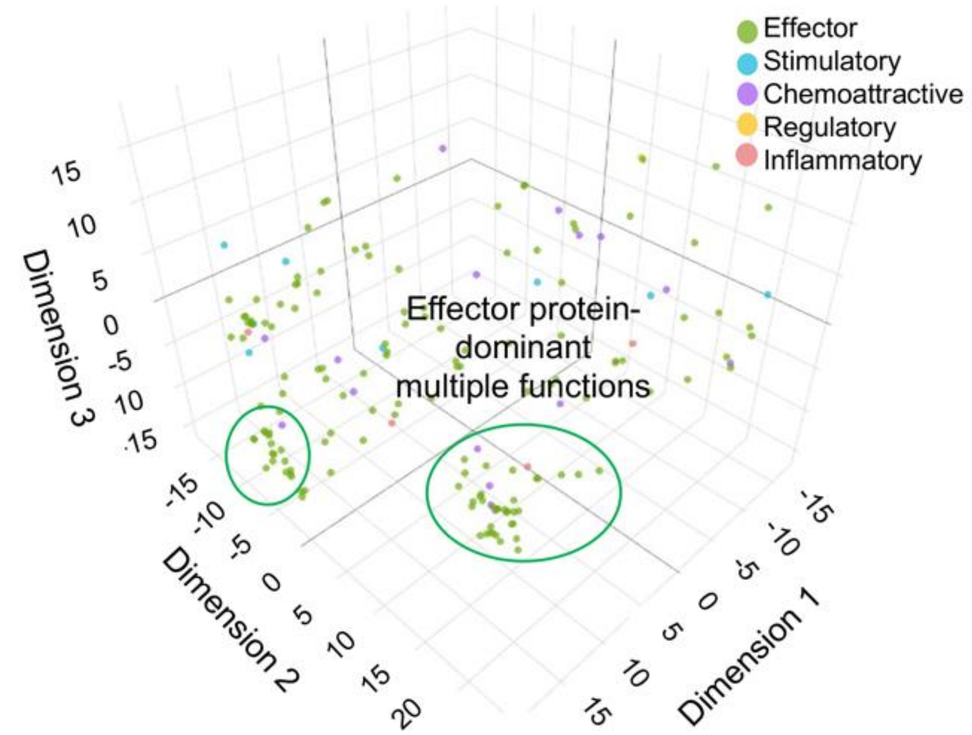

Supplementary Fig. 11: The t-distributed stochastic neighbor embedding (tSNE) plots. The cytokine/chemokines released from polyfunctional long-persistent CD4$^+$ B7-H3 CAR-T cells mainly belonged to the effector subgroup using a 32-plex panel polyfunctionality capture platform.

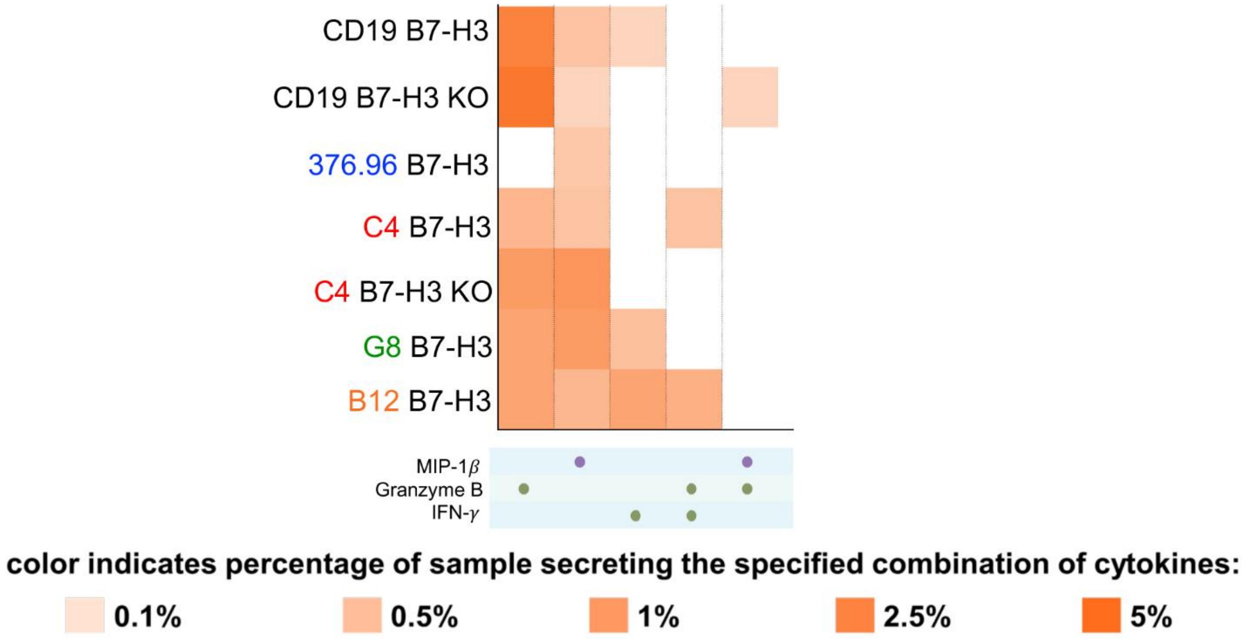

Supplementary Fig. 12: Polyfunctional heat map displaying major function cytokines/chemokines secreted across long-persistent CD8^{+} B7-H3 CAR-T cells upon IMR32 and IMR32 B7-H3 KO stimulation from Fig. 7a.

Supplementary Table 1: The amino acid sequences of B12, C4, and G8 V_HHs.

Name	Sequence	Patent Application
B12 V_HH	QVQLVESGGGSVQVGGSLRLSCAASGFTYNSYSVGWFRQAPGKEREGVAAINSGGSSTYYAASVKGRFTISRDNAKNTVYLQMNSLKPEDTAMYYCAARSPSPLTFQTRTLREDSYNYWGQGTQVTVSS	PCT/US2020/056601
C4 V_HH	EVQLVESGGGSVQAGGSLRLSCVASEDSTSAMCMGWFRQAPGKEREGVACINPTGEVTWYGDSVKGRFTISRDTVKKIVYLQMNSLKPEDTAMYYCAARVTYGGDWSTDTDYEYWGQGTQVTVSS	PCT/US2020/056601
G8 V_HH	DVQLVESGGGLVQPGGSLRLSCAASGFTFSRYWMGWFRQAPGKGVEWVSTINSGGGSTYYADSVKGRFTISRDNAKNTLYLQLNNLKTEDTAMYYCAKEQWRTGSRGQGTQVTVSS	PCT/US2020/056601

Supplementary Table 2: List of 32 genes significantly enriched in low and high polyfunctionality subsets of B12(V_HH)-CAR-T cells. The DESeq2, and R library, was used for the differential expression analysis test.

Gene	Base Mean	Log2 Fold Change	P Value	Functional Cluster	Major Function
EPRS1	1	-0.45	0.011888	**High**	Peptide chain elongation
ATP5PB	0.8	-0.42	0.018614	**High**	ATP synthesis
TUBA1C	1.2	-0.41	0.023464	**High**	Cytoskeleton organization
RBM39	1.4	-0.41	0.023889	**High**	mRNA splicing/transcriptional coactivator
EIF1AX	1.3	-0.37	0.038974	**High**	Translation initiation
AGO3	0.6	-0.37	0.030522	**High**	Transcription repression
MRPL52	1.2	-0.37	0.041478	**High**	Mitochondrial translation
CBWD1	0.5	-0.36	0.033645	**High**	ATP binding
KRT10	0.5	-0.34	0.049954	**High**	Keratinization
FTX	0.4	-0.32	0.048403	**High**	X-inactivation
CHST12	1.8	0.58	0.00124	**Low**	Sulfate transfer (metabolism)
NDUFA1	1.9	0.48	0.004035	**Low**	Electron transfer from NADH to the respiratory chain
NDUFS7	0.7	0.44	0.014092	**Low**	Electron transfer from NADH to the respiratory chain
MFSD4B	0.4	0.39	0.01999	**Low**	Glucose transporter
ACTG1	2.7	0.38	0.01443	**Low**	Cell motility
YWHAB	1.7	0.37	0.030401	**Low**	Signal transduction by binding to phosphoserine-containing proteins
JPX	0.7	0.37	0.038301	**Low**	X-inactivation
TCEAL4	0.7	0.37	0.041	**Low**	Transcription elongation

C19orf53	1.5	0.36	0.040371	**Low**	Undecided but diseases associated with C19orf53 include Leydig Cell Tumor
RPL26L1	0.7	0.36	0.042514	**Low**	Peptide chain elongation
MRPS16	0.9	0.36	0.043855	**Low**	Protein synthesis within the mitochondrion
CSE1L	0.6	0.35	0.04865	**Low**	Export receptor for importin-alpha from nucleus to cytoplasm
PPIB	2	0.35	0.032289	**Low**	Collagen formation
STMN1	3.5	0.34	0.016097	**Low**	Regulation of the microtubule filament system by destabilizing microtubules
RPL30	6.7	0.33	0.005861	**Low**	Peptide chain elongation
RAN	3.5	0.31	0.031645	**Low**	Translocation of RNA and proteins through the nuclear pore complex and control of DNA synthesis and cell cycle progression
COX7A2	4.3	0.29	0.031153	**Low**	Electron transfer from reduced cytochrome c to oxygen
CHCHD2	4.7	0.29	0.027479	**Low**	Peroxisomal lipid metabolism
RPL34	10.2	0.25	0.011286	**Low**	Peptide chain elongation
RPS27	15.6	0.18	0.034877	**Low**	Peptide chain elongation and DNA repair as well as oncogenesis
RPL35	15.7	0.18	0.03556	**Low**	Peptide chain elongation
RPS18	36.3	0.14	0.032758	**Low**	Peptide chain elongation

Supplementary Table 3: Reactome pathway analysis of genes that are significantly enriched in low and high polyfunctionality subsets of B12(V_HH)-CAR-T cells. A hypergeometric distribution test, which is corrected for false discovery rate using the Benjamani-Hochberg method, was performed in this study.

Gene Pathway	P value	Genes
Translation	0.002311816	*MRPL52;EPRS1;EIF1AX*
Formation of the cornified envelope	0.006374501	*KRT10*
Post-transcriptional silencing by small RNAs	0.006624202	*AGO3*
Competing endogenous RNAs (ceRNAs) regulate PTEN translation	0.007567253	*AGO3*
Regulation of PTEN mRNA translation	0.00850949	*AGO3*
Small interfering RNA (siRNA) biogenesis	0.00850949	*AGO3*
Regulation of RUNX1 Expression and Activity	0.01601816	*AGO3*
Formation of ATP by chemiosmotic coupling	0.0169531	*ATP5PB*
Keratinization	0.017234755	*KRT10*
Microtubule-dependent trafficking of connexons from Golgi to the plasma membrane	0.018820558	*TUBA1C*
Transport of connexons to the plasma membrane	0.019753076	*TUBA1C*
Post-chaperonin tubulin folding pathway	0.021615697	*TUBA1C*
Cytosolic tRNA aminoacylation	0.022545801	*EPRS1*
MicroRNA (miRNA) biogenesis	0.022545801	*AGO3*
Formation of tubulin folding intermediates by CCT/TriC	0.024403597	*TUBA1C*
Prefoldin mediated transfer of substrate to CCT/TriC	0.026258182	*TUBA1C*
Activation of AMPK downstream of NMDARs	0.027184272	*TUBA1C*
Cristae formation	0.029034051	*ATP5PB*
Regulation of MECP2 expression and activity	0.029957741	*AGO3*
RHO GTPases activate IQGAPs	0.029957741	*TUBA1C*

Sealing of the nuclear envelope (NE) by ESCRT-III	0.029957741	*TUBA1C*
Cooperation of Prefoldin and TriC/CCT in actin and tubulin folding	0.030880631	*TUBA1C*
Organelle biogenesis and maintenance	0.031168556	*TUBA1C;ATP5PB*
Oncogene Induced Senescence	0.031802724	*AGO3*
NR1H3 & NR1H2 regulate gene expression linked to cholesterol transport and efflux	0.035483124	*AGO3*
Gap junction assembly	0.035483124	*TUBA1C*
Transcriptional Regulation by VENTX	0.036401234	*AGO3*
tRNA Aminoacylation	0.039150801	*EPRS1*
tRNA modification in the nucleus and cytosol	0.040065737	*EPRS1*
Aggrephagy	0.040979881	*TUBA1C*
Assembly and cell surface presentation of NMDA receptors	0.040979881	*TUBA1C*
Carboxyterminal post-translational modifications of tubulin	0.042805795	*TUBA1C*
NR1H2 and NR1H3-mediated signaling	0.044628548	*AGO3*
Gap junction trafficking	0.045538741	*TUBA1C*
Recycling pathway of L1	0.045538741	*TUBA1C*
Gap junction trafficking and regulation	0.047356762	*TUBA1C*
Formation of the ternary complex, and subsequently, the 43S complex	0.048264591	*EIF1AX*
COPI-independent Golgi-to-ER retrograde traffic	0.049171634	*TUBA1C*
Intraflagellar transport	0.050077891	*TUBA1C*

Supplementary Table 4: Antibody information in this study.

Antibodies	Source	Identifier	Dilution	Application
B12	This study	N/A	1-10 μg/ml	ELSIA/Flow cytometry/Western blot
C4	This study	N/A	1-10 μg/ml	ELSIA/Flow cytometry/Western blot
G8	This study	N/A	1-10 μg/ml	ELSIA/Flow cytometry/Western blot
Anti-B7-H3 mAb	Abcam	ab226256	1:1000	Western blot
Anti-B7-H3 mAb	Cell Signaling Technology	14058	1:1000	Western blot
Anti-B7-H3 mAb, clone 376.96	Millipore Sigma	MABC1731-100UL	1-5 μg/ml	ELSIA/Flow cytometry/Western blot
Recombinant humanized anti-human CD276 antibody, clone MGA271	Creative Biolabs	TAB-117CL	1 μg/ml	Flow cytometry
Rabbit anti-human GAPDH	Cell Signaling Technology	2118	1:1000	Western blot
BV711-conjuagted CD3	BioLegend	300464	5 μg/sample	Flow cytometry
FITC-conjugated anti-CD8	BioLegend	344704	5 μg/sample	Flow cytometry
APC/Cy7-conjugated anti-CD4	BioLegend	317417	5 μg/sample	Flow cytometry
APC-conjugated anti-PD1	BioLegend	329908	5 μg/sample	Flow cytometry
APC-conjugated anti-LAG3	Thermo Fisher Scientific	17-2239-41	5 μg/sample	Flow cytometry
APC-conjugated anti-CD62L	BioLegend	304809	5 μg/sample	Flow cytometry
BV421-conjugated anti-TIM3	BioLegend	345007	5 μg/sample	Flow cytometry
BV421-conjugated anti-CD45RA	BioLegend	304130	5 μg/sample	Flow cytometry
PE-conjugated anti-CD95	BioLegend	305608	5 μg/sample	Flow cytometry
PE-conjugated anti-PD-1	Thermo Fisher Scientific	12-2799-42	5 μg/sample	Flow cytometry

PE-conjugated anti-TIM-3	Thermo Fisher Scientific	12-3109-42	5 μg/sample	Flow cytometry
PE-conjugated anti-LAG-3	Thermo Fisher Scientific	12-2239-41	5 μg/sample	Flow cytometry
Cleaved Caspase-3	Cell Signaling Technology	9661	1:500	Western blot
PE-conjugated goat anti-mouse IgG	Jackson ImmunoResearch	115-116-072	1:500	Flow cytometry
APC-conjugated goat anti-mouse IgG	Jackson ImmunoResearch	115-136-072	1:200	Flow cytometry
PE-conjugated goat anti-human IgG	Jackson ImmunoResearch	109-116-097	1:200	Flow cytometry
Alexa Fluor 488-conjugated goat-anti-human IgG	Jackson ImmunoResearch	109-545-003	1:200	Flow cytometry
HRP-conjugated goat anti-human IgG	Jackson ImmunoResearch	109-035-008	1:5000	Western blot
HRP-conjugated goat anti-mouse IgG	Jackson ImmunoResearch	115-035-008	1:5000	Western blot
APC-conjugated -anti-FLAG	Biolegend	637308	1:100	Flow cytometry
HRP-conjugated mouse anti-M13	GE Healthcare	Discontinued	1:5000	ELISA
HRP-conjugated mouse anti-M13	SinoBiological	11973-MM05T-H	1:5000	ELISA
HRP-conjugated mouse anti-His	GenScript	A00612	1:3000	ELISA
Anti-EGFR human monoclonal antibody cetuximab (Erbitux)	Eli Lilly	N/A	1 μg/ml	Flow cytometry

Supplementary Table 5: The amino acid sequence of the 4IgB7-H3 peptides. Each peptide is 18 amino acids long and has 9 overlapped amino acids with adjacent peptides.

Name	Sequence	Name	Sequence
peptide 1	LEVQVPEDPVVALVGTDA	peptide 19	TTSQMANEQGLFDVHSIL
peptide 2	VVALVGTDATLCCSFSPE	peptide 20	GLFDVHSILRVVLGANGT
peptide 3	TLCCSFSPEPGFSLAQLN	peptide 21	RVVLGANGTYSCLVRNPV
peptide 4	PGFSLAQLNLIWQLTDTK	peptide 22	YSCLVRNPVLQQDAHSSV
peptide 5	LIWQLTDTKQLVHSFAEG	peptide 23	LQQDAHSSVTITPQRSPT
peptide 6	QLVHSFAEGQDQGSAYAN	peptide 24	TITPQRSPTGAVEVQVPE
peptide 7	QDQGSAYANRTALFPDLL	peptide 25	GAVEVQVPEDPVVALVGT
peptide 8	RTALFPDLLAQGNASLRL	peptide 26	DPVVALVGTDATLRCSFS
peptide 9	AQGNASLRLQRVRVADEG	peptide 27	DATLRCSFSPEPGFSLAQ
peptide 10	QRVRVADEGSFTCFVSIR	peptide 28	LNLIWQLTDTKQLVHSFT
peptide 11	SFTCFVSIRDFGSAAVSL	peptide 29	TKQLVHSFTEGRDQGSAY
peptide 12	DFGSAAVSLQVAAPYSKP	peptide 30	KDLRPGDTVTITCSSYRG
peptide 13	QVAAPYSKPSMTLEPNKD	peptide 31	TITCSSYRGYPEAEVFWQ
peptide 14	SMTLEPNKDLRPGDTVTI	peptide 32	EQGLFDVHSVLRVVLGAN
peptide 15	LRPGDTVTITCSSYQGYP	peptide 33	GTYSCLVRNPVLQQDAHG
peptide 16	TCSSYQGYPEAEVFWQDG	peptide 34	PVLQQDAHGSVTITGQPM
peptide 17	EAEVFWQDGQGVPLTGNV	peptide 35	SVTITGQPMTFPPEA
peptide 18	QGVPLTGNVTTSQMANEQ		

Supplementary Table 6: Sequences of primers used for B7-H3 and β-actin amplifications.

Name	Sequence (5’ to 3’)
Human B7-H3-forward	GTGGTTCTGCCTCACAGGAG
Human B7-H3-reverse	ACCAGCAGTGCAATGAGACA
Human β-actin-forward	CACCAACTGGGACGACAT
Human β-actin-reverse	ACAGCCTGGATAGCAACG
