# Supplementary material for: Camel nanobody-based B7-H3 CAR-T cells show high efficacy against large solid tumours
Source: Nat Commun. 2023 Sep 22;14:5920. doi: 10.1038/s41467-023-41631-w (PMC10517151; doi:10.1038/s41467-023-41631-w)
Supplement: Supplementary file 3 — Reporting Summary [file 41467_2023_41631_MOESM3_ESM.pdf]

nature portfolio

Corresponding author(s): Mitchell Ho

Last updated by author(s): 08/19/2023

Reporting Summary

Nature Portfolio wishes to improve the reproducibility of the work that we publish. This form provides structure for consistency and transparency in reporting. For further information on Nature Portfolio policies, see our Editorial Policies and the Editorial Policy Checklist.

Statistics

For all statistical analyses, confirm that the following items are present in the figure legend, table legend, main text, or Methods section.

n/a	Confirmed	
☐	☒	The exact sample size (n) for each experimental group/condition, given as a discrete number and unit of measurement
☐	☒	A statement on whether measurements were taken from distinct samples or whether the same sample was measured repeatedly
☐	☒	The statistical test(s) used AND whether they are one- or two-sided<br>*Only common tests should be described solely by name; describe more complex techniques in the Methods section.*
☒	☐	A description of all covariates tested
☐	☒	A description of any assumptions or corrections, such as tests of normality and adjustment for multiple comparisons
☐	☒	A full description of the statistical parameters including central tendency (e.g. means) or other basic estimates (e.g. regression coefficient) AND variation (e.g. standard deviation) or associated estimates of uncertainty (e.g. confidence intervals)
☐	☒	For null hypothesis testing, the test statistic (e.g. F, t, r) with confidence intervals, effect sizes, degrees of freedom and P value noted<br>*Give P values as exact values whenever suitable.*
☒	☐	For Bayesian analysis, information on the choice of priors and Markov chain Monte Carlo settings
☒	☐	For hierarchical and complex designs, identification of the appropriate level for tests and full reporting of outcomes
☒	☐	Estimates of effect sizes (e.g. Cohen's d, Pearson's r), indicating how they were calculated

Our web collection on statistics for biologists contains articles on many of the points above.

Software and code

Policy information about availability of computer code

Data collection	SoftMax Pro 7; FortéBio 8.0; LEGENDplex 8.0; Image Lab 2.0; Living Image 4.5.4; BD FACSDiva 6.0; RCutadapt version 3.4; Alevin Salmon version 1.4.0; RossettaFold; AlphaFold v2.0
Data analysis	Prism 9; RTsne v. 0.15; EDSeq2 v 1.30; R pheatmap library v. 1.0.12; Reactome Pathway Browser v 3.7

For manuscripts utilizing custom algorithms or software that are central to the research but not yet described in published literature, software must be made available to editors and reviewers. We strongly encourage code deposition in a community repository (e.g. GitHub). See the Nature Portfolio guidelines for submitting code & software for further information.

Data

Policy information about availability of data

All manuscripts must include a data availability statement. This statement should provide the following information, where applicable:

- Accession codes, unique identifiers, or web links for publicly available datasets
- A description of any restrictions on data availability
- For clinical datasets or third party data, please ensure that the statement adheres to our policy

Raw and processed data from the scRNA sequencing experiments are deposited, publicly released and available in the NCBI's Gene Expression Omnibus (GEO) database under accession code GSE221411 (https://www.ncbi.nlm.nih.gov/geo/query/acc.cgi?acc=GSE221411). The remaining data are available within the Article, Supplementary Information or Source Data files.

Human research participants

Policy information about studies involving human research participants and Sex and Gender in Research.

Reporting on sex and gender	N/A
Population characteristics	N/A
Recruitment	N/A
Ethics oversight	N/A

Note that full information on the approval of the study protocol must also be provided in the manuscript.

Field-specific reporting

Please select the one below that is the best fit for your research. If you are not sure, read the appropriate sections before making your selection.

☒ Life sciences ☐ Behavioural & social sciences ☐ Ecological, evolutionary & environmental sciences

For a reference copy of the document with all sections, see nature.com/documents/nr-reporting-summary-flat.pdf

Life sciences study design

All studies must disclose on these points even when the disclosure is negative.

Sample size	Sample sizes for in vivo experiments, e.g. treatment cohorts, were used on the basis of ensuring results obtained were of a representable quantity. A population range of 3-8 mice per group was used to ensure statistical power. For in vitro studies, sample size of at least 3 was used in each experiment. Sample size and number of independent experiments are stated in the figure legends.
Data exclusions	No data points was excluded to draw conclusions.
Replication	Three replicates were successfully performed to ensure data reproducibility.
Randomization	Once mice were confirmed to carry tumors, they were randomized into different treatment arms/cages. Cell culture experiments were also randomly assigned to different groups.
Blinding	Investigators were blind for randomization.

Reporting for specific materials, systems and methods

We require information from authors about some types of materials, experimental systems and methods used in many studies. Here, indicate whether each material, system or method listed is relevant to your study. If you are not sure if a list item applies to your research, read the appropriate section before selecting a response.

Materials & experimental systems

n/a	Involved in the study	
☐	☒	Antibodies
☐	☒	Eukaryotic cell lines
☒	☐	Palaeontology and archaeology
☐	☒	Animals and other organisms
☒	☐	Clinical data
☒	☐	Dual use research of concern

Methods

n/a	Involved in the study	
☒	☐	ChIP-seq
☐	☒	Flow cytometry
☒	☐	MRI-based neuroimaging

Antibodies

Antibodies used	Anti-B7-H3 mAb (Abcam, Cat#ab226256, 1:1000 dilution)<br>Anti-B7-H3 mAb (Cell Signaling Technology, Cat#14058, 1:1000 dilution)<br>Anti-B7-H3 mAb, clone 376.96 (Millipore Sigma, Cat#MABC1731-100UL, 1 µg/sample)<br>Recombinant humanized anti-human CD276 antibody, clone MGA271 (Creative Biolabs, Cat# TAB-117CL, 1 µg/ml)<br>Three anti-B7-H3 nanobodies were produced in our lab: B12-Fc, C4-Fc, and G8-Fc (1-10 µg /ml)

Rabbit anti-human GAPDH mAb (Cell Signaling Technology, Cat#2118, 1:1000 dilution)
BV711-conjuagted CD3 (BioLegend, Cat#300464, 5 μl/sample)
FITC-conjugated anti-CD8 (BioLegend, Cat#344704, 5 μl/sample)
APC/Cy7-conjugated anti-CD4 (BioLegend, Cat#317417, 5 μl/sample)
APC-conjugated anti-PD1 (BioLegend, Cat#329908, 5 μl/sample)
APC-conjugated anti-LAG3 (Thermo Fisher Scientific, Cat#17-2239-41, 5 μl/sample)
APC-conjugated anti-CD62L (BioLegend, Cat#304809, 5 μl/sample)
BV421-conjugated anti-TIM3 (BioLegend, Cat#345007, 5 μl/sample)
BV421-conjugated anti-CD45RA (BioLegend, Cat#304130, 5 μl/sample)
PE-conjugated anti-CD95 (BioLegend, Cat#305608, 5 μl/sample)
PE PD-1(Thermo Fisher Scientific, Cat#12-2799-42, 5 μl/sample)
PE TIM-3 (Thermo Fisher Scientific, Cat#12-3109-42, 5 μl/sample)
PE LAG-3 (Thermo Fisher Scientific, Cat#12-2239-41, 5 μl/sample)
Cleaved Caspase-3 (Cell Signaling Technology, Cat#9661, 1:500 dilution)
Goat anti-mouse IgG conjugated with phycoerythrin (PE) (Jackson ImmunoResearch, Cat#115-116-072, 1:200 dilution)
Goat anti-mouse IgG conjugated with allophycocyanin (APC) (Jackson ImmunoResearch, Cat#115-136-072, 1:200 dilution)
Goat anti-human IgG conjugated with PE (Jackson ImmunoResearch, Cat#109-116-097, 1:200 dilution)
Goat-anti-human IgG conjugated with Alexa Fluor 488 (Jackson ImmunoResearch, Cat#109-545-003, 1:200 dilution)
IgG-HRP-conjugated goat anti-human IgG (Jackson ImmunoResearch, Cat#109-035-008, 1:5000 dilution)
IgG-HRP-conjugated goat anti-mouse IgG (Jackson ImmunoResearch, Cat#115-035-008, 1:5000 dilution)
Anti-FLAG antibody conjugated with APC (Biolegend, Cat#637308, 1:100 dilution)
HRP-conjugated mouse anti-M13 antibody (GE Healthcare, 1:5000 dilution)
HRP-conjugated mouse anti-M13 antibody (SinoBiological, Cat#11973-MM05T-H, 1:5000 dilution)
HRP-conjugated mouse anti-His tag antibody (GenScript, 1:3000 dilution)
Anti-EGFR human monoclonal antibody cetuximab (Erbitux) at 1 μg/ml

Validation

Commercial antibodies are all validated by the vendors and the validation data is available on the vendors' website.

Eukaryotic cell lines

Policy information about cell lines and Sex and Gender in Research

Cell line source(s)

Pancreatic cancer cell lines including Panc-1 (originally from ATCC #CRL-1469), T3M4, KLM1, and BxPC-3 (originally from ATCC #CRL-1687) were obtained from Drs. Perwez Hussain, Udo Rudloff, and Christine Alewine at the National Cancer Institute (NCI, Bethesda, MD), respectively. Neuroblastoma cell lines including IMR5, IMR32, NBEB, and LAN-1 were obtained from NCI Pediatric Oncology Branch (Bethesda, MD). Lung cancer cell line M30 was a gift from Dr. Raffit Hassan at the NCI (Bethesda, MD). Triple-negative breast cancer cell line MDA-MB-231, ovarian cancer cell lines NCI-ADR-RES and COV434, lung cancer cell line H226, and epidermoid carcinoma cell line Ca Ski, were purchased from American Type Culture Collection (ATCC). Ovarian cancer cell line OVCAR8 was obtained from National Cancer Institute (Development Therapeutics Program). Lung cancer cell line L55 was provided by Dr. Steven M. Albelda at the University of Pennsylvania (Philadelphia, PA). Two liver cancer (HCC) cell lines Hep3B (#HB-8064), HepG2 (#HB-8065), an epidermoid carcinoma cell line A431 (#CRL-1555), and HEK-293T (#CRL-3216) were purchased from American Type Culture Collection (Manassas, VA). Three lymphoma cell lines including Jurkat (originally from ATCC #TIB-152), Daudi (originally from ATCC #CCL-213), and Raji (originally from ATCC #CCL-86) were gifts from Dr. Ira Pastan in NCI (Bethesda, MD). Luciferase-p2A-mCherry (ML) overexpressed NBEB, IMR32, IMR32 B7-H3 OK, and NBEB B7-H3 OK cell lines were obtained from Brad St Croix at the NCI (Frederick, MD). GFP/Luciferase overexpressed Panc-1, IMR5, BxPC-3, LAN-1, H226, and MDA-MB-231 cell lines were produced in our lab.

Authentication

STR profiling was used for cell line authentication.

Mycoplasma contamination

All cell lines tested negative for mycoplasma contamination.

Commonly misidentified lines
(See ICLAC register)

No commonly misidentified lines were used in the study.

Animals and other research organisms

Policy information about studies involving animals; ARRIVE guidelines recommended for reporting animal research, and Sex and Gender in Research

Laboratory animals

5-7 weeks of female NOD/SCID/IL-2Rgcnull (NSG) mice were used in this study. Mice were housed under a standard 12:12 h light/dark cycle with a standard food and water.

Wild animals

No wild animals were used in this study.

Reporting on sex

The findings are not sex-related.

Field-collected samples

No field-collected samples were used in this study. The findings are based on tumor models in female mice. We have indicated the sex in the Abstract section.

Ethics oversight

The mouse experiments were conducted under the protocol (LMB-059) approved by the Institutional Animal Care and Use Committee at the NIH.

Note that full information on the approval of the study protocol must also be provided in the manuscript.

Flow Cytometry

Plots

Confirm that:

☒ The axis labels state the marker and fluorochrome used (e.g. CD4-FITC).

☒ The axis scales are clearly visible. Include numbers along axes only for bottom left plot of group (a 'group' is an analysis of identical markers).

☒ All plots are contour plots with outliers or pseudocolor plots.

☒ A numerical value for number of cells or percentage (with statistics) is provided.

Methodology

Sample preparation	Samples were stained in 5% BSA in PBS with different antibody dilutions.
Instrument	SONY ID7000, LSR-Fortessa
Software	FlowJo
Cell population abundance	Cell population abundance was determined using counting beads.
Gating strategy	Live cells were gated from FSC/SSC plot, then FSC-A/FAS-H. CAR+(anti-EGFR+anti-IgG-APC)lymphocytes were gated on live cells. CD3+ cells were gated on live cells. T cell exhaustion and differentiation were analyzed in the CD3+CAR+ T cell population.

☒ Tick this box to confirm that a figure exemplifying the gating strategy is provided in the Supplementary Information.
